# The presence of ovarian endometrioma adversely affect ovarian reserve and response to stimulation but not oocyte quality or IVF/ICSI outcomes: a retrospective cohort study

**DOI:** 10.1186/s13048-022-01042-9

**Published:** 2022-10-22

**Authors:** Cheng Zeng, Ruihui Lu, Xin Li, Yanrong Kuai, Sheng Wang, Qing Xue

**Affiliations:** Department of Obstetrics and Gynecology, Peking University First Hospital, 100034 Beijing, P. R. China

**Keywords:** Endometriosis, ART, Ovarian reserve, Ovarian response, Clinical outcomes

## Abstract

**Background:**

The possible impact of ovarian endometriomas (OMAs) on in vitro fertilization (IVF) outcomes remains controversial. Therefore, this study aimed to assess the impact of OMAs on IVF cycle parameters, including ovarian reserve and response to stimulation, embryo quality and pregnancy outcomes.

**Methods:**

This retrospective cohort study included 2067 patients undergoing their first IVF/ICSI cycles between January 2018 and December 2020. The study group included 154 infertile women who had OMAs. The control group consisted of 1913 women without endometriosis, and finally 305 women were matched according to maternal age, body mass index (BMI), and infertility duration by propensity score matching (PSM). Cumulative live birth rate (CLBR) was set as the primary outcome measure. Logistic regression analysis was conducted on the basis of clinical covariates assessed for their association with CLBRs. Subgroup analyses were performed to evaluate the effect of ovarian surgery, cyst size and laterality on CLBRs.

**Results:**

Women with OMAs had significantly lower ovarian reserve markers (AMH and AFC), number of follicles, oocytes, embryos, and top-quality embryos than women in the control group (p < 0.05). However, the CLBRs were comparable between the two groups (55.64% versus 54.34%, p = 0.806), regardless of previous history of ovarian surgery. Multivariate analysis revealed association between age (OR = 0.861; 95% CI [0.806–0.921]; p = 0.000), top-quality embryos (OR = 1.829; 95% CI [1.526–2.193]; p = 0.000) and the CLBRs. A negative correlation between OMA size and AFC levels in patients with unoperated OMAs was detected (r = -0.264, p = 0.007). Meanwhile, significant decrease in ovarian reserve with lower AFC, fewer oocytes, embryos and top-quality embryos were observed in patients with OMAs size ≥ 6 cm (p < 0.05). Moreover, ovaries with OMAs had a significantly lower AFC (P = 0.006) but similar number of oocytes when compared with contralateral ovaries without OMAs.

**Conclusion:**

Infertile women with OMAs were implicated in considerable decreases in ovarian reserve and response to stimulation, but no apparent adverse effects on oocyte quality or clinical outcomes. OMAs surgery and OMAs size may adversely affect ovarian reserve, but not CLBR.

**Supplementary Information:**

The online version contains supplementary material available at 10.1186/s13048-022-01042-9.

## Introduction

Endometriosis (EMS), characterized by the development of endometrial-like tissue in aberrant locations outside the uterine cavity, is a chronic estrogen-dependent disease with an overall prevalence of 10–15% in women of reproductive age and up to 50% in infertile women [1, 2]. The symptoms of pelvic endometriosis, that is painful periods, painful intercourse, and chronic pelvic pain and infertility, often disrupt the social, professional, academic, and economic potential of reproductive age women.

Despite its dependency on sex steroid hormones and inflammation, the exact pathogenesis of endometriosis on infertility remains poorly understood. Treatments for infertility caused by endometriosis mainly include surgery and assisted reproductive technology (ART) [3]. Surgery has been shown to enhance the chances of conceiving naturally during the 12–18 ensuing months irrespective of the stage of the disease [4]. However, surgery is of no advantage when ART is considered, as it does not improve pregnancy outcome but carries the risk of decreasing ovarian reserve and further responses to ovarian stimulation [4, 5]. Therefore, ART is commonly the primary option to be considered in women whose infertility is associated with ovarian endometriomas (OMAs) with a mean diameter below 40 mm and whose ovarian reserve is compromised and/or who are over 35 years of age [6].

The possible impact of ovarian endometriosis on ART results remains a controversial issue. Many studies have elucidated a detrimental effect of ovarian endometriosis on oocyte quality, embryo quality and/or endometrial receptivity [7, 8]. Meanwhile, single-cell RNA sequencing of oocytes from patients with endometriosis has identified dysregulated mechanisms involved in steroid metabolism and biosynthesis, response to oxidative stress and cell cycle regulation [9]. Thus, dysregulation of these mechanisms, which reduce oocyte quality, raises concerns for decreased ART outcomes in patients with OMAs. Consistent with this, lower implantation and pregnancy rates were observed in mild and severe endometriosis patients compared to other causes of infertility [10] or healthy women [11], indicating a poorer success in the in vitro fertilization/ intracytoplasmic sperm injection (IVF/ICSI) outcomes with an increase in severity of the disease. Moreover, ovarian reserve evaluated with serum antimullerian hormone (AMH) or antral follicle count (AFC) is reduced in patients with OMAs compared to patients with other benign ovarian cysts, or to patients without OMAs [12, 13]. However, several meta-analyses indicated that women with OMAs undergoing IVF/ICSI had similar reproductive outcomes compared with those without the disease, although the cycle cancellation rate was significantly higher [14], and number of oocytes retrieved was reduced [15]. Therefore, the aim of the present study was to evaluate the IVF/ICSI outcomes in patients with visual OMAs at the start of stimulated cycles in comparison to that in patients without endometriosis. That is, to assess the impact of OMAs on IVF cycles parameters, especially on ovarian reserve and response to stimulation, embryo quality and IVF/ICSI outcomes.

## Methods

### Patient population and management

This was a retrospective observational cohort study of 2067 patients undergoing their first IVF/ICSI cycles with autologous oocytes, conducted at the reproductive medicine center of Peking University First Hospital (China) between January 2018 and December 2020. The study protocol was reviewed by the institutional ethics review board of Peking University First Hospital.

The study group included 154 women who had one or more OMAs in unilateral or bilateral ovaries at the start of stimulated cycles. The diagnosis of endometriosis was confirmed by ultrasound, Magnetic Resonance Imaging (MRI), or following abdomen-pelvic surgery with histologically confirmed ovarian endometriosis. Women with OMAs had undergone imaging (i.e., ultrasound and/or MRI) that resulted in a recognizable ovarian cyst according to the usual criteria, including size, appearance, cyst content, unilocular or multilocular, vascularization, and the presence of vegetation [16]. For each patient, the cyst laterality (i.e., left, right, or bilateral) and size (in millimeters) were recorded. The cyst size was based on the largest diameter as determined by ultrasound-based imaging. In case of bilateral cysts, the sum of the largest diameters and the sum of the volumes of each cyst were considered for analysis when appropriate [17].

For comparison, the control group consisted of 1913 women who underwent IVF treatment during the same time period, without any evidence of ovarian endometriosis or prior history of surgery for endometriosis. As groups were not randomly assigned, potential confounders and selection biases were accounted for by propensity score matching (PSM) [18]. A 1:2 nearest neighbor matching method without replacement was performed to match data between women with OMAs and the control group with a caliper width equal to 0.02. The matched variables included maternal age, maternal BMI, and duration of infertility.

Exclusions were due to treatment using donor eggs; with hydrosalpinx, intrauterine adhesion, uterine structural malformation, fibroids diameter ≥ 40 mm, polycystic ovarian syndrome, systemic lupus erythematosus or other rheumatologic disease; use of hormonal medications or hormonal or non-hormonal anti-inflammatory agents during the 3 months prior to inclusion in this study or if aimed at fertility preservation. Patient baseline characteristics, including age, body mass index (BMI), AFC, baseline follicle-stimulating hormone (FSH), luteinizing hormone (LH), and estradiol (E2) levels, were collected from the database. The baseline hormone levels were obtained on the third day of the menstrual cycle, one to three months before the treatment cycle.

### Controlled ovarian stimulation and IVF/ICSI procedure

A flexible long gonadotropin-releasing hormone (GnRH) agonist protocol or an antagonist protocol were used for controlled ovarian stimulation. The GnRH agonist long protocol consisted of daily injections of short-acting GnRH agonist and of long-acting GnRH agonist at different doses during the early follicular or mid-luteal phases. The choice of protocol for ovarian stimulation was based on the patient’ s characteristics. Human chorionic gonadotropin (hCG) was administered when more than two follicles reached a diameter greater than 18 mm on ultrasound. Transvaginal ultrasound-guided oocyte retrieval was performed 36 h after hCG administration by single-lumen needle aspiration. After retrieval, oocytes were fertilized by standard insemination. ICSI was performed only in cases with severe male factor infertility or previous fertilization failure. Embryos were cultured for up to six days. ASEBIR embryo assessment criteria and Gardner’s classification were used to assess embryo morphology at the cleavage stage and blastocyst stage with minor modification [19]. Only one or two embryos were transferred, depending on patient age, cycle rank, and embryo quality, but irrespective of the endometriosis status of the patient. Fresh embryo transfer was generally performed on day 2 or day 3 after the oocyte retrieval. All remaining good quality embryos were cryopreserved by vitrification, according to the manufacturer’s recommendations. Frozen–thawed embryo transfer (FET) was performed through three main types of endometrial preparation protocols: the natural cycle (NC), hormone replacement treatment (HRT) cycle with or without GnRH downregulation. The luteal supported phase was administered by vaginal or intramuscular administration of progesterone until 8 weeks after embryo transfer (ET). If the pregnancy test was positive, luteal phase support was continued until the 10th gestational week.

### Outcome measures

Biological pregnancy was initially diagnosed by a serum hCG level above 100 IU/L, which was tested 12–14 days after ET according to the embryo stage. Clinical pregnancy was confirmed by the presence of a gestational sac on vaginal ultrasound examination during the fourth week after ET. A live birth was defined as birth event with at least one baby born alive (> 24 weeks of gestation). Ovarian sensitivity was calculated as the ovarian sensitivity index (OSI), defined as number of oocytes retrieved divided by the total dose of gonadotrophins administered * 1,000 [20]. Cumulative live birth rate was defined as the rate of live birth following the transfer of all (fresh or frozen-thawed) embryos available from the stimulated cycle [21]. Only the first delivery was considered in the analysis.

Cumulative live birth rate (CLBR) was set as the primary outcome measure. The secondary outcome measures were AMH, AFC, OSI, the number of oocytes retrieved, the metaphase stage II (MII) oocytes retrieved, the maturity rate, the number of embryos, the fertilization rate, the proportion of top-quality embryos, the proportion of transplantable embryos, and the number of frozen embryos, the implantation rate, the clinical pregnancy rate (CPR) and the live birth rate (LBR).

To evaluate the effect of ovarian surgery on IVF/ICSI outcomes of patients with OMAs, the study group was further divided into two subgroups according to their history of ovarian surgery (categorized as follows: (A) OMA without prior ovarian cyst surgery; (B) OMA with a prior history of ovarian cyst surgery), and the relative clinical variables were analyzed and compared. Subgroup analyses were also performed according to the cyst size (categorized as follows: (A) OMAs diameter < 40 mm; (B) OMAs diameter ≥ 40 mm and < 60 mm; (C) OMAs diameter ≥ 60 mm) or cyst laterality (i.e., the presence of unilateral or bilateral OMAs).

### Statistical analysis

Data analyses were performed using SPSS 22 package program (SPSS Inc, Chicago, IL, USA). Categorical data were presented as the numbers and percentages. Continuous variables were given as the mean ± standard deviation (SD) and were tested using Student’s t-test or non-parametric Mann-Whitney U-test. To compare qualitative variables, chi-square and Fisher’ s exact tests were used as indicated. PSM was conducted to select the control cohort by using MatchIt package in R software (version 3.6.2). A 1:2 nearest neighbor matching method without replacement was conducted with a caliper width equal to 0.02. Logistic regression analysis was performed to determine the variables that could be independently associated with CLBRs per OPU. Multivariable logistic regression (MLR) was performed on variables that were significant at univariable analysis (P < 0.05). Odds ratios (OR) and their 95% confidence intervals (CI) were calculated from the model’ s coefficients and their standard deviations. A spearman’s correlation was run to assess the relationship between the count of AFC and OMAs cyst volume. All analyses of significance were 2-sided, and a value of P < 0.05 was considered statistically significant.

## Results

A total of 2067 women were enrolled in this study. The endometriosis group consisted of 154 patients with visual OMAs at the time of OPU (the OMAs group). The control group included 1913 patients without visual endometriosis and no prior surgery for OMAs. After PSM, the 154 women with OMAs were matched by age, BMI and duration of infertility at a 1:2 ratio to the 305 control women. For the OMAs group, the specific endometriosis phenotype was as follows: 120 (77.92%) had unilateral OMAs, while 34 (22.08%) had bilateral OMAs. The mean size of the OMA lesions was 36.80 ± 24.39 mm. OMAs diameter < 40 mm was found in 107/154 (69.48%) of these women and OMAs diameter ≥ 60 mm was found in 20/154 (12.99%) of these women. Lastly, 50 (32.25%) of patients in the OMAs group had previously undergone surgery for OMAs.

Patients’ overall demographics and baseline IVF characteristics were presented in Table 1 (left panel). Significant differences were observed in terms of BMI, duration of infertility, AMH, AFC, basal FSH, ovarian stimulation protocol, total Gn administered and number of follicles ≥ 10 mm on day of hCG between the two groups (P < 0.05). Comparison after PSM was also listed in Table 1 (right panel). No significant differences were observed between the two groups in regard to age, BMI, and duration of infertility, showing a valid matching in the enrolled population (P > 0.05). Patients in the OMAs group had significantly lower ovarian reserve markers, with a significantly lower mean serum AMH level (2.47 ± 2.34 ng/mL vs. 3.32 ± 2.82 ng/mL, p = 0.001) and AFC (8.08 ± 5.22 ng/mL vs. 12.29 ± 7.66 ng/mL, p = 0.000). However, basal serum FSH, LH and E2 levels were comparable between the two groups after matching (P > 0.05).

**Table 1:** Baseline characteristics and ovarian stimulation parameters in the endometrioma and control groups

Variable	Before matching			After matching		
	**Endometrioma (OMA)** **(n = 154)**	**Control** **(n = 1913)**	**P value**	**Endometrioma (OMA)** **(n = 154)**	**Control** **(n = 305)**	**P value**
**Baseline characteristics**						
Age (years)	33.55 ± 3.82	33.68 ± 4.93	0.694	33.55 ± 3.82	33.65 ± 5.03	0.812
BMI (kg/m^2^)	21.66 ± 2.85	22.61 ± 4.19	**0.000**	21.66 ± 2.85	21.96 ± 2.93	0.293
Duration of infertility (years)	2.86 ± 2.09	3.33 ± 2.70	**0.010**	2.86 ± 2.09	2.70 ± 1.90	0.396
Type of infertility						
Primary n (%)	91 (59.09)	1162 (60.74)	0.852	91 (59.09)	187 (61.31)	0.646
Secondary n (%)	63 (40.91)	751 (39.26)		63 (40.91)	118 (38.69)	
AMH (ng/mL)	2.47 ± 2.34	3.32 ± 2.96	**0.000**	2.47 ± 2.34	3.32 ± 2.82	**0.001**
AFC (n)	8.08 ± 5.22	12.26 ± 7.41	**0.000**	8.08 ± 5.22	12.29 ± 7.66	**0.000**
Basal FSH (IU/L)	10.03 ± 6.72	8.90 ± 5.00	**0.044**	10.03 ± 6.73	8.88 ± 4.76	0.063
Basal LH (IU/L)	4.85 ± 2.42	5.08 ± 4.44	0.519	4.85 ± 2.42	5.25 ± 4.51	0.305
Basal E2 (pg/ml)	98.01 ± 270.39	124.91 ± 509.70	0.518	98.01 ± 270.39	132.75 ± 494.13	0.422
**Ovarian stimulation parameters**						
Stimulation Protocol						
Agonist n (%)	64 (41.56)	652 (34.08)	**0.030**	64 (41.56)	90 (29.51)	**0.010**
Antagonist n (%)	70 (45.45)	1076 (56.25)		70 (45.45)	184 (60.33)	
Natural or mild stimulation n (%)	20 (12.99)	185 (9.67)		20 (12.99)	31 (10.16)	
Duration of stimulation (days)	9.66 ± 2.56	10.03 ± 2.41	0.070	9.66 ± 2.56	9.89 ± 2.37	0.348
Total Gn administered (IU)	2958.93 ± 1141.20	2704.04 ± 1118.38	**0.007**	2958.93 ± 1141.20	2643.20 ± 1086.46	**0.004**
E2 on day of hCG (pg/ml)	2683.47 ± 2131.08	3013.27 ± 1989.37	0.051	2683.47 ± 2131.08	3090.01 ± 1948.96	**0.046**
Progesterone on day of hCG (ng/ml)	1.10 ± 0.50	1.37 ± 9.08	0.712	1.10 ± 0.50	1.19 ± 0.75	0.115
Endometrial thickness on day of hCG (mm)	10.85 ± 2.51	10.55 ± 2.50	0.147	10.85 ± 2.51	10.45 ± 2.46	0.100
Number of follicles ≥ 10 mm on day of hCG	11.06 ± 7.92	13.62 ± 10.13	**0.002**	11.06 ± 7.92	13.64 ± 9.17	**0.002**
Cycle cancellation rate % (n)	1.30 (2/154)	1.46 (28/1913)	1.000	1.30 (2/154)	0.66 (2/305)	0.605

BMI, body mass index; AMH, anti-Müllerian hormone; AFC, Antral Follicular Count; E2, estradiol; FSH, follicle-stimulating hormone; Gn, gonadotropin; hCG, human chorionic gonadotropin; OSI, Ovarian sensitivity index. Values are expressed as n (%), percentage (%) or mean ± standard deviation (SD) unless otherwise stated. P-values in bold depict statistical significance (P < 0.05).

The agonist protocol was more often prescribed in the OMAs group than in the control group (41.56% vs. 34.08%, 41.56% vs. 29.51%, respectively) before and after matching (P = 0.030, P = 0.010, respectively). Women with OMAs required significantly greater doses of Gn administered (2958.93 ± 1141.20 vs. 2643.20 ± 1086.46, P = 0.004) but a lower number of follicles on day of hCG than in the control group (11.06 ± 7.92 vs. 13.64 ± 9.17, P = 0.002). Meanwhile, the number of oocytes retrieved (8.27 ± 6.18 vs. 10.25 ± 6.97, p = 0.005) and the OSI (3.23 ± 3.07 vs. 4.93 ± 4.82, P = 0.000) of women with OMAs were significantly lower than those in the control group (Table 2), indicating a decreased ovarian response to stimulation among women with OMAs.

**Table 2:** Embryological data and IVF/ICSI outcomes in the endometrioma and control groups

Variable	Before matching			After matching		
	**Endometrioma** **(OMA)** **(n = 154)**	**Control** **(n = 1913)**	**P value**	**Endometrioma** **(OMA)** **(n = 154)**	**Control** **(n = 305)**	**P value**
**Embryological data**						
Number of oocytes retrieved (n)	8.27 ± 6.18	9.99 ± 6.44	**0.002**	8.27 ± 6.18	10.25 ± 6.97	**0.005**
OSI	3.23 ± 3.07	4.52 ± 4.06	**0.000**	3.23 ± 3.07	4.93 ± 4.82	**0.000**
Number of MII oocytes (n)	6.99 ± 5.41	8.00 ± 5.67	**0.034**	6.99 ± 5.41	8.33 ± 5.97	**0.040**
Oocyte maturity rate % (n)	84.57 (1063/1257)	80.11 (15084/18829)	**0.000**	84.57 (1063/1257)	80.74 (2523/3125)	**0.003**
Number of fertilized oocytes (n)	6.20 ± 5.04	7.20 ± 5.24	**0.025**	6.20 ± 5.04	7.45 ± 5.54	**0.032**
Fertilization rate % (n)	83.30 (943/1132)	84.98 (13562/15959)	0.128	83.30 (943/1132)	85.36 (2256/2643)	0.108
Number of Embryos (n)	6.34 ± 5.10	7.32 ± 5.30	**0.028**	6.34 ± 5.10	7.47 ± 5.58	**0.038**
Number of transplantable embryos (n)	5.69 ± 4.78	6.63 ± 4.88	**0.022**	5.69 ± 4.78	6.72 ± 5.02	**0.035**
Number of top-quality embryos (n)	2.32 ± 2.48	2.73 ± 2.74	0.078	2.32 ± 2.48	2.82 ± 3.01	**0.038**
Frozen embryos	2.95 ± 3.31	3.03 ± 3.34	0.773	2.95 ± 3.31	3.16 ± 3.42	0.575
Blastocyst rate % (n)	28.53 (269/943)	27.59 (3742/13562)	0.535	28.53 (269/943)	27.84 (628/2256)	0.692
Type of embryo transfer						
Total ET cycles	128 (83.12%)	1831 (97.14%)	**0.000**	128 (83.12%)	293 (96.07%)	**0.000**
Fresh ET n (%)	49 (38.28)	884 (48.28)	**0.029**	49 (38.28)	138 (47.10)	0.094
FET n (%)	79 (61.72)	947 (51.72)		79 (61.72)	155 (52.90)	
Stage of embryo transfer						
Cleavage (n)	110	1568	0.925	110	231	0.088
Blastocyst (n)	18	263		18	62	
**Clinical outcomes**						
CPRs / fresh ET % (n)	55.10 (27/49)	45.14 (399/884)	0.173	55.10 (27/49)	46.37 (64/138)	0.294
CPRs / FET % (n)	53.16 (42/79)	54.20 (510/941)	0.860	53.16 (42/79)	66.45 (103/155)	**0.048**
LBRs / fresh ET % (n)	48.98 (24/49)	37.00 (327/884)	0.092	48.98 (24/49)	36.96 (51/138)	0.140
LBRs / FET % (n)	48.10 (38/79)	45.38 (427/941)	0.501	48.10 (38/79)	59.35 (92/155)	0.101
Miscarriages % (n)	4.69 (6/128)	7.34 (134/1825)	0.373	4.69 (6/128)	7.17 (21/293)	0.339
ectopic pregnancy % (n)	0.78 (1/128)	1.04 (19/1825)	1.000	0.78 (1/128)	1.02 (3/293)	1.000
Implantation rate % (n)	38.56 (91/236)	34.80 (1196/3437)	0.241	38.56 (91/236)	39.67 (217/547)	0.770
Cumulative CPRs % (n)	59.40 (79/133)	58.12 (981/1688)	0.773	59.40 (79/133)	59.62 (158/265)	0.966
Cumulative LBRs % (n)	55.64 (74/133)	53.26 (899/1688)	0.596	55.64 (74/133)	54.34 (144/265)	0.806

FET, Frozen-thawed ET; MII, Metaphase II; CPRs, Clinical pregnancy rates; LBRs, Live birth rates. Values are presented as n, n (percentage), or the mean ± standard deviation (SD). Dashes indicate no P-value. P-values in bold depict statistical significance (P < 0.05).

Overall, the embryological data and IVF/ICSI treatment outcomes were summarized in Table 2. After matching, the number of MII oocytes was significantly lower in women with OMAs than in the control group (6.99 ± 5.41 vs. 8.33 ± 5.97, p = 0.040), as were the number of fertilized oocytes (6.20 ± 5.04 vs. 7.45 ± 5.54, p = 0.032), total embryos (6.34 ± 5.10 vs. 7.47 ± 5.58, p = 0.038), transplantable embryos (5.69 ± 4.78 vs. 6.72 ± 5.02, p = 0.035) and top-quality embryos (2.32 ± 2.48 vs. 2.82 ± 3.01, p = 0.038). However, the fertilization rate and the blastocyst rate were not significantly different between the two groups. Embryo transfer was achieved for 83.12% (128/154) of the women in the OMAs group and for 96.07% (293/305) of the women in the control group (p = 0.000). Similar proportions of cleavage or blastocyst embryos were transferred in both groups (p = 0.088).

In fresh cycles, the CPRs did not differ between women with OMAs vs. Controls (55.10% vs. 46.37%, p = 0.294). The LBRs were also similar between the two groups (48.98% vs. 36.96%, p = 0.140). In FET cycles, LBRs were similar between the OMAs and the control group (48.10% vs. 59.35%, p = 0.101), but CPRs was lower in women with OMAs than in the control group (53.16% vs. 66.45%, p = 0.048). The primary outcome measures, the cumulative CPRs (59.40% vs. 59.62%, p = 0.966) and the CLBRs (55.64% vs. 54.34%, p = 0.806) showed no significant differences between the OMAs and the control group.

The results of the univariate and multivariate analysis of factors affecting the CLBRs in patients undergoing IVF/ICSI were presented in Table 3. As regards to comparison between endometriosis vs. other infertility diagnosis, the results of univariate analysis showed a significantly lower CLBRs in patients with diminished ovarian reserve (DOR) compared with endometriosis (OR = 0.159; 95% CI: 0.069–0.367; p = 0.000), but no differences between endometriosis vs. tubal factor infertility, male factor infertility, anovulation, and unspecified infertility cause. However, in multivariate analysis, CLBRs was similar and did not differ significantly between women with endometriosis vs. DOR (p = 0.087). Meanwhile, age (OR = 0.836), AMH (OR = 1.318), AFC (OR = 1.099), follicles on day of hCG (OR = 1.113), OSI (OR = 1.290) and top-quality embryos (OR = 2.135) were associated with a significantly difference in CLBRs in univariate analysis. After multivariate analysis, age (OR = 0.861) and top-quality embryos (OR = 1.829) remained independent factors associated with CLBRs. In addition, AMH, AFC, stimulation protocol, OSI, number of follicles were not significantly associated with an increased CLBRs.

**Table 3:** Logistic regression analysis of factors affecting the cumulative live-birth rates per cycle in IVF/ICS patients

	Univariate analysis		Multivariate analysis	
**variables**	**OR (95%CI)**	**P value**	**OR (95%CI)**	**P value**
Infertility diagnosis				
Endometrioma	Reference	-		
Tubal factor	1.172 (0.579–2.372)	0.658		
Male factor	1.163 (0.624–2.166)	0.635		
Anovulation	1.993 (0.728–5.454)	0.179		
DOR	0.159 (0.069–0.367)	**0.000**	0.380 (0.125–1.153)	0.087
Unspecified	1.495 (0.864–2.588)	0.151		
Age	0.836 (0.792–0.882)	**0.000**	0.861 (0.806–0.921)	**0.000**
BMI	0.969 (0.903–1.039)	0.371		
AMH	1.318 (1.188–1.462)	**0.000**	0.987 (0.831–1.173)	0.885
AFC	1.099 (1.063–1.136)	**0.000**	0.994 (0.936–1.055)	0.837
Stimulation Protocol				
Agonist n (%)	Reference	-		
Antagonist n (%)	0.362 (0.227–0.578)	**0.000**	0.774 (0.403–1.487)	0.442
Natural or mild stimulation n (%)	0.143 (0.066–0.308)	**0.000**	1.456 (0.425–4.984)	0.550
Number of follicles on day of hCG (≥ 10 mm)	1.113 (1.081–1.145)	**0.000**	0.996 (0.949–1.046)	0.875
OSI	1.290 (1.190–1.399)	**0.000**	1.009 (0.897–1.136)	0.876
Number of top-quality embryos	2.135 (1.802–2.529)	**0.000**	1.829 (1.526–2.193)	**0.000**
Type of embryo transfer				
Fresh ET (n)	Reference			
FET (n)	1.561 (0.993–2.456)	0.054		

OR, odds ratio; CI, Confidence Interval; DOR, diminished ovarian reserve; BMI, body mass index; AMH, anti-Müllerian hormone; AFC, Antral Follicular Count; Gn, gonadotropin; hCG, human chorionic gonadotropin; OSI, Ovarian sensitivity index. Dashes indicate no P-value. P-values in bold depict statistical significance.

Next, in order to assess the impact of prior OMA surgery on the ovarian reserve and response, as well as IVF/ICSI outcomes, patients were further divided into two subgroups according to their previous history of ovarian surgery (Table 4). Among them, 104 had OMAs without prior ovarian surgery and 50 had OMAs and a history of prior cyst surgery. When compared to the OMAs without surgery group, patients with a history of prior cyst surgery had significantly lower ovarian reserve parameters (AMH and AFC), poor ovarian response parameters (OSI and number of follicles), thus resulted in significantly lower numbers of matured and fertilized oocytes and embryos. However, the oocyte maturity rate, fertilization rate and implantation rate were comparable within the two groups. Meanwhile, we observed a slight trend towards a lower proportion of CLBRs (50.00% vs. 57.14%) in patients with previous history of ovarian surgery, but the difference did not reach clinical significance when compared with those who had OMAs without previous surgery.

**Table 4:** IVF cycle characteristics and outcomes in endometriosis patients with and without prior surgery

Variable	Endometrioma without surgery (n = 104)	Endometrioma with surgery (n = 50)	P value
Age (years)	33.92 ± 4.08	32.88 ± 3.18	0.114
BMI (kg/m^2^)	21.48 ± 2.86	22.00 ± 2.83	0.293
Duration of infertility (years)	2.85 ± 2.04	2.97 ± 2.22	0.754
AMH (ng/mL)	2.93 ± 2.68	1.61 ± 1.13	**0.000**
AFC (n)	8.68 ± 5.41	6.92 ± 4.54	**0.048**
Basal FSH (IU/L)	10.25 ± 7.5448	9.85 ± 5.65	0.747
Duration of stimulation (days)	9.66 ± 2.68	9.72 ± 2.37	0.899
Total Gn administered (IU)	2865.99 ± 1111.65	3198.75 ± 1227.20	0.095
E2 on day of hCG (pg/ml)	3071.55 ± 2425.97	2167.69 ± 1537.13	**0.016**
Number of follicles ≥ 10 mm on day of hCG	11.73 ± 8.95	7.76 ± 6.33	**0.002**
Number of oocytes retrieved (n)	9.28 ± 6.74	6.20 ± 4.19	**0.001**
OSI	3.80 ± 3.48	2.06 ± 1.40	**0.000**
Number of MII oocytes (n)	7.75 ± 5.90	5.44 ± 3.86	**0.004**
Oocyte maturity rate % (n)	83.53 (791/947)	87.74 (272/310)	0.075
Number of fertilized oocytes (n)	6.92 ± 5.49	4.74 ± 3.59	**0.004**
Fertilization rate (%)	81.68 (682/835)	78.45 (233/297)	0.225
Number of Embryos (n)	7.02 ± 5.61	4.96 ± 3.50	**0.006**
Number of transplantable embryos (n)	6.35 ± 5.35	4.57 ± 3.33	**0.013**
Number of top-quality embryos (n)	2.53 ± 2.59	1.98 ± 2.22	0.200
Implantation rate % (n)	35.33 (59/167)	43.84 (32/73)	0.211
Cumulative CPRs % (n)	58.24 (53/91)	59.52 (25/42)	0.889
Cumulative LBRs % (n)	57.14 (52/91)	50.00 (21/42)	0.442

BMI, body mass index; AMH, anti-Müllerian hormone; AFC, Antral Follicular Count; E2, estradiol; FSH, follicle-stimulating hormone; Gn, gonadotropin; hCG, human chorionic gonadotropin; OSI, Ovarian sensitivity index; FET, Frozen-thawed ET; MII, Metaphase II, CPRs, Clinical pregnancy rates; LBRs, Live birth rates. Values are expressed as n (%), percentage (%) or mean ± standard deviation (SD) unless otherwise stated. P < 0.05 depicts statistical significance. * depicts P < 0.05 when compared with group (Endometrioma without surgery). ** depicts P < 0.01 when compared with group (Endometrioma without surgery). *** depicts P < 0.001 when compared with group (Endometrioma without surgery). **** depicts P < 0.0001 when compared with group (Endometrioma without surgery).

To examine whether the IVF cycle characteristics and outcomes correlate with the size of OMAs, we performed a subgroup analysis based on patients with confirmed OMAs but without previous ovarian surgery (n = 104, Table 5). These patients were divided into three group according to the size of OMAs (categorized as follows: (A) OMAs diameter < 40 mm; (B) OMAs diameter ≥ 40 mm and < 60 mm; (C) OMAs diameter ≥ 60 mm). The baseline characteristics, ovarian stimulation and IVF/ICSI outcomes in our study were not significantly different in women with an OMA diameter ≥ 40 mm and < 60 mm as compared to those with OMAs diameter < 40 mm. However, women with OMAs diameter ≥ 60 mm had a significant lower AFC (3.85 ± 3.31 vs. 9.56 ± 5.31, p = 0.000), fewer follicles on the day of HCG (6.92 ± 7.94 vs. 12.67 ± 9.01, p = 0.034), fewer oocytes retrieved (5.08 ± 5.14 vs. 10.17 ± 7.08, p = 0.012) than those in women with OMAs diameter < 40 mm, indicating a decreased ovarian reserve. Likewise, number of fertilized oocytes (p = 0.011), embryos (p = 0.017), transplantable embryos (p = 0.011) and top-quality embryos (p = 0.037) were also lower in women with OMAs diameter ≥ 60 mm when compared to the OMAs diameter < 40 mm group. As regard to IVF/ICSI outcomes, a trend towards a lower CLBRs was observed in the OMAs diameter ≥ 60 mm group compared with OMAs diameter < 40 mm group (41.67%, vs. 58.73%, p = 0.275), but no significant difference was achieved. The result of spearman’s correlation showed that there was a negative correlation between OMA size and AFC levels in patients with unoperated OMAs (r = -0.264, p = 0.007).

**Table 5:** IVF cycle characteristics and outcomes in patients with unoperated OMAs according to the cyst size

Variable	Endometrioma size < 40 mm (n = 72)	Endometrioma size ≥ 40 and < 60 mm (n = 18)	Endometrioma size ≥ 60 mm (n = 14)	P value
Age (years)	33.64 ± 3.89	33.33 ± 3.53	35.23 ± 3.92	0.324
BMI (kg/m^2^)	21.56 ± 2.71	20.89 ± 23.19	21.58 ± 3.25	0.671
Duration of infertility (years)	2.82 ± 1.84	3.14 ± 2.82	2.77 ± 2.01	0.830
AMH (ng/mL)	3.18 ± 2.86	2.38 ± 1.66	2.19 ± 2.81	0.332
AFC (n)	9.56 ± 5.31	8.94 ± 5.42	3.85 ± 3.31 ****	**0.002**
Basal FSH (IU/L)	9.22 ± 5.73	12.44 ± 11.89	12.92 ± 7.73	0.114
Basal E2 (pg/ml)	86.50 ± 201.05	96.15 ± 135.79	51.77 ± 40.58	0.772
Duration of stimulation (days)	10.01 ± 2.23	9.33 ± 3.41	8.39 ± 3.52	0.107
Total Gn administered (IU)	3020.49 ± 1071.67	2668.75 ± 1090.75	2284.62 ± 1248.48*	0.064
E2 on day of hCG (pg/ml)	3265.05 ± 2596.00	3084.31 ± 2015.99	1589.90 ± 1626.60*	0.132
Number of follicles ≥ 10 mm on day of hCG	12.67 ± 9.01	11.33 ± 8.94	6.92 ± 7.94*	0.103
Number of oocytes retrieved (n)	10.17 ± 7.08	8.77 ± 5.13	5.08 ± 5.14 *	**0.039**
OSI	4.13 ± 3.89	3.51 ± 2.16	2.34 ± 1.83	0.220
Number of MII oocytes (n)	8.46 ± 6.28	7.41 ± 4.23	4.31 ± 4.44*	0.062
Oocyte maturity rate % (n)	83.20 (609/732)	84.56 (126/149)	84.85 (56/66)	0.879
Number of fertilized oocytes (n)	7.60 ± 5.84	6.77 ± 4.09	3.39 ± 3.57 *	**0.037**
Fertilization rate (%)	82.32 (517/628)	86.49 (96/111)	69.49 (41/59) *	**0.020**
Number of embryos (n)	7.63 ± 5.99	7.12 ± 3.98	3.54 ± 4.05*	0.052
Number of transplantable embryos (n)	6.94 ± 5.58	6.47 ± 3.69	2.92 ± 3.86 *	**0.038**
Number of top-quality embryos (n)	2.79 ± 2.82	2.53 ± 2.03	1.15 ± 1.21*	0.112
Implantation rate % (n)	35.67 (56/157)	34.78 (8/23)	50 (8/16)	0.566
Cumulative CPRs % (n)	60.32 (38/63)	62.50 (10/16)	41.67 (5/12)	0.452
Cumulative LBRs % (n)	58.73 (37/63)	62.50 (10/16)	41.67 (5/12)	0.490

BMI, body mass index; AMH, anti-Müllerian hormone; AFC, Antral Follicular Count; E2, estradiol; FSH, follicle-stimulating hormone; Gn, gonadotropin; hCG, human chorionic gonadotropin; OSI, Ovarian sensitivity index; FET, Frozen-thawed ET; MII, Metaphase II, CPRs, Clinical pregnancy rates; LBRs, Live birth rates. Values are expressed as n (%), percentage (%) or mean ± standard deviation (SD) unless otherwise stated. P < 0.05 depicts statistical significance. * depicts P < 0.05 when compared with group (Endometrioma size < 40). ** depicts P < 0.01 when compared with group (Endometrioma size < 40). *** depicts P < 0.001 when compared with group (Endometrioma size < 40). **** depicts P < 0.0001 when compared with group (Endometrioma size < 40).

**Table 6:** IVF characteristics in ovaries with and without endometrioma in patients with unoperated unilateral OMAs.

Variable	Ovary with endometrioma (n = 80)	Contralateral ovary (n = 80)	P value
AFC (n)	3.80 ± 2.86	4.90 ± 3.15	**0.006**
Number of follicles ≥ 10 mm on day of hCG	5.11 ± 4.32	5.47 ± 4.88	0.544
Number of oocytes retrieved (n)	3.97 ± 3.20	4.37 ± 3.53	0.355

AFC, Antral Follicular Count; hCG, human chorionic gonadotropin. Values are expressed as mean ± standard deviation (SD) unless otherwise stated. P < 0.05 depicts statistical significance.

Furthermore, another subgroup analysis was performed according to the laterality of OMAs (bilateral or unilateral), and the results were showed in Table S1. AMH, AFC, number of follicles on day of hCG, oocytes retrieved, MII oocytes, fertilized oocyte and embryos were lower in patients with bilateral OMAs than those in patients with unilateral OMAs, but no significant difference was found. In consequence, the IVF/ICSI outcomes (i.e. CPRs, LBRs and CLBRs) were not significantly different in women with bilateral OMAs as compared to unilateral OMAs. Moreover, comparisons between ovaries with OMAs and the contralateral ovaries in women with unoperated unilateral OMAs are presented in Table 6. Ovaries with OMAs had a significantly lower AFC (3.80 ± 2.86 vs. 4.90 ± 3.15, P = 0.006) but a similar number of follicles on day of hCG (5.11 ± 4.32 vs. 5.47 ± 4.88, P = 0.544) and oocytes retrieved (3.97 ± 3.20 vs. 4.37 ± 3.53, P = 0.355) when compared with the contralateral ovaries.

## Discussion

The present study investigated the baseline characteristics, ovarian stimulation parameters, embryological data and clinical outcomes of IVF/ICSI treatment in patients with and without endometriosis. The results demonstrated that the ovarian reserve and response to stimulation for IVF/ICSI treatment was significantly lower in patients with OMAs compared with non-endometriosis controls after adjusting for age, BMI, and infertility duration with PS matching. However, the clinical pregnancy outcomes, especially cumulative CPRs and LBRs, did not differ significantly between the two groups. Moreover, the results of MLR indicated that the CLBRs was highly associated with age and number of top-quality embryos. Notably, statistical difference was not achieved when considering the impact of ovarian surgery and the OMAs size on CLBRs in endometriosis patients, although a significantly diminished ovarian reserve and poor response to stimulation were observed. This cohort study also demonstrated that, compared with patients with unilateral OMAs, patients with bilateral OMAs had lower AMH and AFC, fewer number of matured and fertilized oocytes, and lower proportion of cumulative CPRs and LBRs, but there were no significant differences. Interestingly, we found that ovaries with OMAs had a significantly lower AFC but a similar number of follicles and oocytes retrieved when compared with the contralateral ovaries in patients with unilateral OMAs.

Emerging evidence suggest that endometriosis is detrimental to the ovaries [1, 7]. Since the toxic content from an OMA may lead to unfavorable events such as increased oxidative stress, increase fibrosis, loss of cortex specific stroma, smooth muscle cell metaplasia, vascularization defect and, later, reduced follicular maturation [14]. In this study, women with endometrioma had a significantly lower ovarian reserve and response compared to the control group, regardless of whether they had any previous ovarian surgery, which was in accordance with previous studies [12, 13, 22]. Conversely, one study [17] reported that preoperative serum AMH levels were found to be the same for women with OMAs and women with a non-OMAs benign cyst. They also found that serum AMH levels positively correlated with the OMAs size regardless of the presence of bilateral OMAs or associated DIE. According to their study, the possible reason why serum AMH levels are higher with large OMAs is that the increased size of OMA promoted secretion of AMH into the circulation by the ovaries. Thus, a lower number of oocytes retrieval could be due to insufficient follicular stimulation with insufficient gonadotropin doses in relation to the serum AMH levels. In our study, the serum AMH levels were significantly lower in infertile women with OMAs compared to the non-endometriosis controls, which is in consistent with a recent study published by Wu et al. [23]. Although we observed a trend towards a lower AMH level as the OMAs size increased, the difference did not reach clinical significance. Instead, a negative correlation between OMAs size and AFC levels in patients with unoperated OMAs was detected. Thus, further studies are needed to explore the correlation between the size of OMAs and the AMH level, not limited to women with infertility.

The impact of endometriosis on oocyte quality and embryo development is still controversial. The diminished ovarian reserve and lower number of oocytes retrieved in women with OMAs compared with women without endometriosis somehow verify the hypothesis that the OMAs per se exert some detrimental impact on the ovary [14]. Recipients of oocyte donors with endometriosis achieved lower pregnancy rates than those who received oocytes from non-endometriosis donors [24]. In addition, Kitajima et al. [25] found that ovaries affected by OMAs present premature follicle recruitment, higher rates of atresia, and lower quality of remaining primordial follicles, which may be related to a intraovarian inflammatory environment. Meanwhile, analysis of the follicular fluid of endometriosis infertile women revealed that excessive reactive oxygen species (ROS) in endometriosis granulosa cells (GCs) induced GCs senescence, which significantly correlated with oocyte retrieval number and mature oocyte number of endometriosis patient [26]. Altogether, these results suggested that endometriosis could impair oocyte microenvironment with deleterious consequences on oocyte and embryo quality. In this study, we found that women with endometriosis had a significantly lower number of oocytes retrieved and mature oocytes despite receiving higher Gn doses, and a significantly lower number of embryos and top-quality embryos, which was consistent with the results of several prior reports [23, 27]. Multivariate analysis conducted by Boucret et al. did not reveal any association between endometriosis and embryo quality [28], and concluded that endometriosis lowers the CLBRs in IVF by decreasing the number of embryos but not their quality. It is worth noting that despite having a significant decrease in the number of oocytes and embryos, we did not find any detrimental impact of endometriosis on oocyte matured, fertilized and blastocyst rate. Thus, further study concerning the embryo development data of unilateral endometrioma is needed.

The possible impact of ovarian endometriosis on IVF/ICSI outcomes remain a controversial issue, with some studies confirming a significant negative impact [10, 28] and others reporting no effect regardless of whether the women had any ovarian surgical history [14, 15, 29]. A large retrospective study included 39,356 IVF cycles in women with endometriosis concluded that women with isolated endometriosis had similar or higher LBRs compared to women with other diagnoses, whereas endometriosis women with concomitant diagnoses had lower LBRs compared with other causes [30]. Consistent with this study, Feichtinger et al. [29] found significantly reduced CLBRs in women with tubal factor compared to endometriosis-related infertility, with the conclusion that a diagnosis of endometriosis, with or without present OMAs, does not negatively affect ART cumulative results. Wu et al. observed lower CLBRs in OMAs women compared with matched non-OMAs women but MLR analysis showed no correlation between OMAs per se and live birth [23]. In our study, pregnancy outcomes were quite comparable between women with OMAs and the controls, regardless of whether the women had any ovarian surgical history, which is in agreement with previous studies indicating that surgical resection of OMA cysts decrease ovarian reserve but have similar outcomes to other patients following ART [5, 14]. Notably, women with OMAs received less total ET cycles than the controls which is attributable due to the fact that number of oocytes retrieved and embryos were lower in patients with OMAs.

Previous studies have mostly focused on the effect of surgical removal of OMAs on ART outcomes rather than the effect of the OMAs itself, thus, we conducted subgroup analyses according to the size and laterality of OMA without previous ovarian surgery. According to the newly published third edition of guideline for the diagnosis and treatment of endometriosis in China, surgical resection of OMAs may be considered when endometriomas diameter ≥ 40 mm. It is worth noting that pregnancy outcomes did not differ between endometriomas < 40 mm and endometriomas ≥ 40 and < 60 mm. Thus, the necessary of surgical resection of OMAs < 60 mm should be considered carefully. Karadag et al. [31]discovered that increased OMA size is related to decreased AMH levels in patients with OMAs and bilateral OMAs have a more destructive effect on ovarian reserve. Moreover, OMAs (size < 60 mm) have no impact on embryo quality or final IVF outcomes (pregnancy and birth rates), despite a possible reduction in the number of oocytes retrieved and potentially higher Gn doses [32]. However, the paper contains no data on OMAs ≥ 60 mm. In our study, a decrease in ovarian reserve with lower AFC, fewer oocytes, embryos and top-quality embryos were observed in patients with endometrioma size ≥ 60 mm, compared with patients with endometrioma size < 40 mm. Although a trend towards a lower CLBRs were observed in endometrioma size ≥ 60 mm, no significant difference was achieved, partly due to the small sample size in this group. Likewise, despite having an obvious trend towards a lower CLBRs, this study failed to show significant difference in pregnancy outcomes between women with bilateral OMAs and those with unilateral OMAs.

Interestingly, we found that ovaries with OMAs had a significantly lower AFC but a similar number of follicles and oocytes retrieved when compared with the contralateral ovaries in patients with unilateral OMAs, which was consistent with prior studies showing that OMAs with diameter ≤ 30 mm do not negatively affect the response to ovarian superovulation [33, 34]. Ovaries with OMAs achieved reduced AFC but similar number of oocytes retrieved, and the authors believed that this is secondary to an impaired ability to detect small follicles in the presence of an OMA. In contrast, Ferrero et al. discovered that the presence of large OMAs (≥ 50 mm) at time of IVF decreases the number of total follicles and oocyte retrieved compared with the contralateral healthy ovaries, with no significant difference in AFC [35]. Considering the small sample size and the retrospectively collected data of these studies, further studies with larger sample size and data on the number and quality of embryos are needed.

A main strength of our study is that PSM was conducted to control the potential confounders which might have effects on the outcomes. PSM provides an approach to mimic random assignment as RCT and is superior to conventional regression-based methods in a real world observational study [18]. Additionally, the comparisons were not only performed in overall groups, but were also explored in patients with and without prior OMA surgery and in women with different characteristics of OMAs (i.e. cyst size and laterality). One crucial limitation is the fact that the control group had undergone no surgery before cycle stimulation. Thus, the presence of endometriosis, especially peritoneal endometriosis, cannot be completely excluded. Meanwhile, our study was limited by its retrospectively observational design. Though PSM was performed to evaluate the effects of OMAs on IVF/ICSI treatment outcomes, the sample size decreased after PSM and the loss of unmatched cases might have unforeseen effects. Thus, further large clinical randomized trials on women with OMAs to examine and compare embryo development and IVF/ICSI outcomes are suggested to further identify the impact of OMAs per se.

## Conclusion

According to the data above, our study demonstrated that infertile women with endometriosis, both with and without a history of prior ovarian surgery, were implicated in considerable decreases in ovarian reserve and response to stimulation, but may have no obvious disadvantages on clinical outcomes. Additionally, a negative correlation between OMA size and AFC levels in patients with unoperated OMAs was detected. Meanwhile, ovaries with OMAs had a significantly lower AFC when compared with the contralateral ovaries. Thus, the present data provides further support for the hypothesis that not only OMAs surgery but also increasing OMAs itself may adversely affect ovarian reserve, but not oocyte quality or IVF/ICSI outcomes. Further large prospective studies are still needed to evaluate whether the presence or the increasing size of OMAs is associated with a worse clinical outcomes.

## Electronic supplementary material

Below is the link to the electronic supplementary material.

Supplementary Material 1

## Data Availability

The datasets used and/or analyzed during the current study are availablefrom the corresponding author on reasonable request.

## Abbreviations

OMAs: Ovarian endometriomas
IVF/ICSI: In vitro fertilization/ intracytoplasmic sperm injection
OPU: Oocyte pickup
PSM: Propensity score matching
BMI: Body mass index
CLBRs: Cumulative live-birth rates
Gn: gonadotropins
EMS: Endometriosis
ART: Assisted reproductive technology
AMH: Antimullerian hormone
AFC: Antral follicle count
MRI: Magnetic Resonance Imaging
PGT: Preimplantation Genetic Testing
FSH: Follicle-stimulating hormone
LH: luteinizing hormone
E2: Estradiol
GnRH: Gonadotropin-releasing hormone
hCG: Human chorionic gonadotropin
FET: Frozen–thawed embryo transfer
HRT: Hormone replacement treatment
NC: Natural cycle
ET: Embryo transfer
OSI: Ovarian sensitivity index
MII: Metaphase stage II
CPR: Clinical pregnancy rate
LBR: Live birth rate
SD: Standard deviation
MLR: Multivariable logistic regression
OR: Odds ratios
CI: Confidence intervals
ROS: Reactive oxygen species
GCs: Granulosa cells
