# Supplementary material for: The presence of ovarian endometrioma adversely affect ovarian reserve and response to stimulation but not oocyte quality or IVF/ICSI outcomes: a retrospective cohort study
Source: J Ovarian Res. 2022 Oct 22;15:116. doi: 10.1186/s13048-022-01042-9 (PMC9587543; doi:10.1186/s13048-022-01042-9)
Supplement: Supplementary file 1 — Supplementary Material 1 [file 13048_2022_1042_MOESM1_ESM.docx]

**Supplemental Table 1. IVF cycle characteristics and outcomes in patients with unilateral and bilateral OMAs without prior surgery.**

| **Variable** | **Unilateral endometrioma (n = 80)** | **Bilateral endometrioma (n = 24)** | **P value** |
| --- | --- | --- | --- |
| Age (years) | 33.76 ± 3.80 | 34.74 ± 4.87 | 0.311 |
| BMI (kg/m^2^) | 21.61 ± 2.90 | 20.83 ± 2.65 | 0.247 |
| Duration of infertility (years) | 2.89 ± 2.14 | 2.83 ± 1.72 | 0.902 |
| AMH (ng/mL) | 3.06 ± 2.76 | 2.43 ± 2.46 | 0.336 |
| AFC (n) | 9.21 ± 5.42 | 7.04 ± 5.16 | 0.091 |
| Basal FSH (IU/L) | 9.99 ± 7.8 | 11.36 ± 6.39 | 0.462 |
| Basal E2 (pg/ml) | 86.93 ± 197.88 | 71.30 ± 52.74 | 0.722 |
| Duration of stimulation (days) | 9.89 ± 2.58 | 9.00 ± 2.95 | 0.163 |
| Total Gn administered (IU) | 2928.91 ± 1050.76 | 2647.83 ± 1325.43 | 0.290 |
| E2 on day of hCG (pg/ml) | 3218 ± 2573.90 | 2573.90 ± 2190.33 | 0.311 |
| Number of follicles ≥10 mm on day of hCG | 12.28 ± 8.84 | 9.74 ± 9.41 | 0.235 |
| Number of oocytes retrieved (n) | 9.67 ± 6.20 | 7.96 ± 8.34 | 0.285 |
| OSI | 3.87 ± 3.15 | 3.54 ± 4.51 | 0.686 |
| Number of MII oocytes (n) | 8.10 ± 5.63 | 6.57 ± 6.74 | 0.274 |
| Number of fertilized oocytes (n) | 7.25 ± 5.14 | 5.78 ± 6.56 | 0.260 |
| Number of embryos (n) | 7.29 ± 5.16 | 6.09 ± 7.00 | 0.368 |
| Number of transplantable embryos (n) | 6.59 ± 4.80 | 5.52 ± 6.58 | 0.393 |
| Number of top-quality embryos (n) | 2.63 ± 2.51 | 2.22 ± 2.88 | 0.507 |
| Implantation rate %(n) | 35.38 (46/130) | 35.48 (11/31) | 0.992 |
| Cumulative CPRs %(n) | 61.97 (44/71) | 45.00 (9/20) | 0.174 |
| Cumulative LBRs %(n) | 60.56 (43/71) | 45.00 (9/20) | 0.214 |

BMI, body mass index; AMH, anti-Müllerian hormone; AFC, Antral Follicular Count; E2, estradiol; FSH, follicle-stimulating hormone; Gn, gonadotropin; hCG, human chorionic gonadotropin; OSI, Ovarian sensitivity index; FET, Frozen-thawed ET; MII, Metaphase II, CPRs, Clinical pregnancy rates; LBRs, Live birth rates. Values are expressed as n (%), percentage (%) or mean ± standard deviation (SD) unless otherwise stated. P<0.05 depicts statistical significance.
